# Representation and Processing of L2 Compositional Multiword Sequences: Effects of Token Frequency, Type Frequency, and Constituency

**DOI:** 10.3390/bs15060734

**Published:** 2025-05-26

**Authors:** Yingying Xu, Yang Yu

**Affiliations:** English Department, School of Foreign Languages, Dalian Maritime University, Dalian 116026, China; yuyang89@dlmu.edu.cn

**Keywords:** compositional multiword sequence, phrase frame, schema, token frequency, type frequency, constituency, L2 proficiency

## Abstract

The present study investigates the effects of token frequency, type frequency, and constituency on L2 compositional multiword sequence (CMS) processing among 60 Chinese L2 English speakers at two proficiency levels, using an online phrasal decision task. The findings reveal the following: (1) Both proficiency groups exhibited a significant token frequency effect in their L2 phrasal and non-phrasal CMS processing, indicating that both sequence types hold psychological reality in L2 learners’ mental representations. (2) The type frequency effect was observed in the higher-proficiency groups’ processing of phrasal and non-phrasal CMSs with low token frequencies, yet it was more pronounced in the less proficient group’s processing of phrasal and non-phrasal CMSs with high token frequencies, indicating that the effect of type frequency operates on a gradient continuum rather than being strictly categorical. (3) Constituency emerged as a robust predictor of processing efficiency, with phrasal CMSs being processed more efficiently than their non-phrasal counterparts across nearly all frequency conditions and proficiency levels. This consistent advantage for phrasal structures underscores the fundamental role of structural integrity in L2 CMS processing. These findings contribute novel insights into the mechanisms underlying L2 CMS processing, while also offering practical pedagogical implications for enhancing L2 CMS acquisition.

## 1. Introduction

Usage-based approaches to language acquisition emphasize the critical role of multiword sequences, or chunks, in language learning. These approaches posit that language acquisition is essentially a process wherein learners, through exposure to specific linguistic items (including a wide range of multiword sequences such as *give me a break*), extract partially schematized slot-and-frame patterns (e.g., *give NP a break*, *give NP NP*), and subsequently generalize these into fully abstract schematic patterns or grammatical structures (e.g., *V NP NP*) (Ellis, 2002; Gries & Ellis, 2015; Wulff, 2019). These patterns, or schemas, which vary in their levels of abstraction, form the basis for linguistic creativity and productivity (Ellis, 2002). Early research on multiword sequence processing predominantly focused on fixed idiomatic expressions (e.g., *beat around the bush*) (Nekrasova, 2009), while more recent corpus-based studies have revealed that natural language use relies heavily on non-idiomatic, compositional multiword sequences (CMSs) (e.g., *at the same time*, *they know what*) (Biber, 2009; Römer, 2009). Those CMSs, whose meanings are derivable from their constituent words, can be classified into two structural types: phrasal sequences (e.g., *make a mistake*), which function as independent syntactic units within a sentence, and non-phrasal sequences (e.g., *an increase in*), which are non-constituent elements of a sentence, such as clausal or phrasal fragments. The present investigation focuses on the processing of both phrasal and non-phrasal CMSs.

Regarding the psychological reality of both phrasal and non-phrasal CMSs, existing research with native speakers and second language (L2) learners has yielded inconclusive findings. While some studies have reported a significant token frequency effect in the processing of phrasal (e.g., Arnon & Snider, 2010; Hernández et al., 2016; Jeong & Jiang, 2019; Supasiraprapa, 2019) and non-phrasal CMSs (e.g., Arnon & Cohen-Priva, 2013; Jolsvai et al., 2013), other investigations have failed to replicate these results, finding no such effect for either phrasal (e.g., Jolsvai et al., 2020; Valsecchi et al., 2013) or non-phrasal CMSs (e.g., Jeong & Jiang, 2019). This inconsistency underscores the complexity of the mental representation of CMSs and highlights the need for a more systematic exploration of their psychological reality. Moreover, although type frequency has been widely acknowledged as a critical factor in language learning and processing (Berg, 2014; Dąbrowska & Szczerbinski, 2006; Gries & Ellis, 2015), its specific role in the processing of multiword sequences remains underexplored. To address this gap, the concept of phrase frames—discontinuous multiword sequences with a single slot (e.g., from his *, where * represents the slot) (Tan & Römer, 2022)—should be introduced. These frames are semi-abstract limited-scope patterns or schemas generalized from multiple CMSs (e.g., *from his home*, *from his office*) (Ren, 2022). While prior research has predominantly focused on the role of token frequency in CMS processing, this study hypothesizes that the processing of CMSs is influenced not only by their token frequency but also by the type frequency of their corresponding phrase frames (see Section 2.1 for details). Additionally, since phrasal and non-phrasal CMSs differ fundamentally in their structural completeness, some scholars suggest that this structural distinction may influence how these recurrent sequences are processed cognitively (Ellis et al., 2008; Nekrasova, 2009; Schmitt et al., 2004). Despite this theoretical proposition, few empirical studies have directly compared the processing of phrasal and non-phrasal sequences by native speakers and L2 learners (e.g., Arnon & Cohen-Priva, 2013; Jeong & Jiang, 2019), and the findings remain inconsistent. This study, therefore, examines the effects of token frequency, type frequency, and constituency on the processing of L2 CMSs by Chinese learners of English at two proficiency levels. Through this investigation, it aims to contribute to a more comprehensive understanding of the cognitive mechanisms underlying the acquisition and processing of L2 CMSs.

## 2. Literature Review

### 2.1. Token Frequency and Type Frequency

In usage-based theories, two distinct yet interrelated frequency measures—token frequency and type frequency—are recognized as fundamental to understanding language acquisition and processing (Bybee, 2008; Ellis, 2002). Given that one of the primary objectives of the present study is to investigate the effects of these frequency measures on L2 CMS processing, it is imperative to first delineate and operationalize these concepts with precision.

Token frequency refers to the number of occurrences of a specific linguistic item (such as a word or CMS) within a given corpus (Bybee, 2008). This measure is widely regarded as a critical determinant of entrenchment, a cognitive process whereby repeated exposure to a linguistic item strengthens its representation in memory, thereby enhancing its automaticity and ease of retrieval (Bybee, 2008). In this study, token frequency is operationalized as the total number of occurrences of a specific CMS within a corpus. For instance, consider the sequences *an attempt to*, *an answer to*, and *an offer to*, with token frequencies of 12,299, 2102, and 756, respectively, in a given corpus. According to usage-based principles (Arnon & Snider, 2010; Bybee, 2013; Supasiraprapa, 2019), *an attempt to* would exhibit the highest degree of psychological entrenchment, followed by *an answer to*, with *an offer to* demonstrating the weakest mental representation. This hierarchy arises because higher token frequency strengthens the associative connections between the components of a sequence, reinforcing its form-meaning mapping in the mental lexicon and facilitating more fluent and accurate retrieval and use (Diessel, 2015).

In contrast, type frequency measures “the number of distinct items that can occur in the open slot of a construction or the number of items that exemplify a pattern” (Bybee, 2008, p. 221). Unlike token frequency, which pertains to specific linguistic items, type frequency is a property of linguistic schemas or patterns and is considered a key determinant of their productivity and generalizability (Bybee & Thompson, 1997; Goldberg, 1995). As the number of distinct items occupying a slot within a pattern increases, the criterial features of the pattern—those shared by its members or instances which restrict the range of items that can fill the slot—become increasingly generalized, thereby expanding its applicability to novel items (Diessel, 2015). In this study, type frequency of a phrase frame is operationalized as the total number of specific CMSs that exemplify the frame. Accordingly, phrase frames with higher type frequencies, which encompass a broader range of specific CMSs, are likely to possess more robust mental representations, making them more readily activated and retrieved for the comprehension and production of novel or low-token-frequency multiword sequences (Bybee, 2013; Wolter & Gyllstad, 2013). For example, consider the phrase frame it is * to, which includes 1700 specific CMSs (e.g., *it is important to*, *it is necessary to*), and the phrase frame to be * that, which includes 300 specific CMSs (e.g., *to be sure that*, *to be certain that*). According to usage-based principles, the former, with a type frequency of 1700, would exhibit a more stable mental representation than the latter, with a type frequency of 300. As a result, for two low-token-frequency CMSs, such as *it is false to* and *to be honest that*, the phrase frame it is * to would be more easily activated and retrieved for the processing of the corresponding low-token-frequency CMS (i.e., *it is false to*).

From a usage-based perspective, the processing of low-token-frequency CMSs is hypothesized to be modulated by type frequency (Bybee, 2013; Wolter & Gyllstad, 2013). However, this proposition raises several theoretically and empirically significant questions that warrant further investigation. First, is the effect of type frequency limited exclusively to low-token-frequency items, or does it extend across a wider range of token frequency bands? Second, is the influence of type frequency contingent upon the structural properties of CMSs, that is, whether they are phrasal or non-phrasal? Third, does the effect of type frequency vary as a function of language users’ proficiency levels? Addressing these questions is crucial for advancing our understanding of the cognitive mechanisms underpinning L2 CMS processing, refining current theoretical models of language acquisition and processing, and informing pedagogical strategies aimed at enhancing L2 CMS acquisition.

### 2.2. Major Factors Influencing CMS Processing

In recent years, a growing body of research has investigated the psychological reality of CMSs and the factors influencing their processing efficiency. This section reviews prior studies examining the effects of token frequency, type frequency, and constituency on CMS processing, highlighting key findings and unresolved issues.

Extensive research on phrasal CMS processing has demonstrated that both native speakers and L2 learners (particularly those at higher proficiency levels) process higher-token-frequency phrases faster and more accurately than lower-token-frequency ones—a phenomenon known as the token frequency effect (e.g., Chen et al., 2023; Hernández et al., 2016; Jeong & Jiang, 2019; N. Jiang & Nekrasova, 2007; S. Jiang & Siyanova-Chanturia, 2023; Tremblay et al., 2011; Schmitt et al., 2004; Supasiraprapa, 2019). While these findings highlight the role of token frequency in phrase processing, other studies have demonstrated more nuanced patterns (e.g., Jolsvai et al., 2020; Valsecchi et al., 2013), suggesting that additional factors may interact with or modulate the influence of token frequency. For instance, in a phrasal decision experiment involving native speakers, Jolsvai et al. (2020) found no significant effects of token frequency in the processing of either idioms (e.g., *play the field*) or compositional phrases (e.g., *get a certificate*). Instead, they observed significant effects of meaningfulness for both types of sequences, with faster decision times for sequences rated as more meaningful. It is understandable that the processing of idioms—a special class of multiword sequences that are often non-compositional and characterized by relatively low token frequencies—may be more influenced by meaning rather than token frequency. The absence of token frequency effects in the processing of compositional phrases, however, is more surprising and warrants more detailed scrutiny. A closer examination of the stimuli employed in Jolsvai et al.’s (2020) study reveals that the compositional phrases exhibited limited variability in both token frequency and meaningfulness, with most phrases characterized by low token frequency paired with high meaningfulness and only a small subset featuring high token frequency with either high or low meaningfulness. This distribution may have influenced the observed outcomes, underscoring the need for a more balanced approach in experimental designs. Therefore, to achieve a more comprehensive understanding of the mechanisms underlying phrasal CMS processing, a more systematic investigation is needed—one that considers additional factors (e.g., L2 proficiency, type frequency) influencing phrasal CMS processing and employs a more balanced experimental design.

In contrast to the extensive research on phrasal CMSs, studies on the processing and mental representation of non-phrasal CMSs remain relatively limited, and existing findings are notably inconsistent. Some studies have documented a token frequency effect in non-phrasal CMS processing, with higher-token-frequency non-phrases being processed more rapidly and accurately than their lower-token-frequency counterparts (e.g., Arnon & Cohen-Priva, 2013; Jolsvai et al., 2013; Tremblay & Baayen, 2010; Tremblay et al., 2011; Yi & Zhong, 2024). For example, Arnon and Cohen-Priva (2013) conducted a production study examining the impact of token frequency on phonetic duration in both elicited and spontaneous speech among native English speakers. They observed similar token frequency effects for phrases (e.g., *a lot of years*) and non-phrases (e.g., *as far as I*): phonetic duration was reduced for higher-token-frequency sequences regardless of constituency. Their results suggest that, like phrases, structurally incomplete non-phrasal CMSs possess psychological reality, at least for native speakers. In contrast, other research has argued that non-phrasal CMSs lack psychological reality (e.g., Jeong & Jiang, 2019; Schmitt et al., 2004). For instance, Jeong and Jiang (2019) compared the processing of phrasal sequences (e.g., *in other words*) and non-phrasal sequences (e.g., *an increase in*) by L1 and L2 English speakers using a word monitoring task. Their results revealed that both participant groups exhibited a significant token frequency effect for phrasal sequences, but not for non-phrasal ones, suggesting that non-phrasal sequences may not be represented in the mental lexicon. Given these conflicting findings and the scarcity of research specifically addressing L2 non-phrasal CMS processing, further investigation into the psychological reality of non-phrasal CMSs in L2 learners is warranted.

Similarly, research on the role of type frequency or phrase frame productivity in CMS processing remains limited. A seminal study by Matthews and Bannard (2010) addressed this gap by examining whether the production of familiar sequences (akin to high-token-frequency sequences, such as *a piece of toast*) and unfamiliar sequences (akin to low-token-frequency sequences, such as *a piece of brick*) in 2- and 3-year-olds was influenced by the productivity of four-gram schematic patterns (or phrase frames, e.g., a piece of *). They found that children in both age groups more readily reproduced unfamiliar sequences with more productive patterns (e.g., out of the *) than those with less productive ones (e.g., Let’s have a *), whereas the production of familiar sequences remained unaffected by schematic productivity. Although not explicitly examining schematic productivity, Wolter and Gyllstad’s (2013) collocational decision task further highlighted the role of abstract patterns in multiword processing. They found that both L1 English speakers and L1 Swedish learners of English made significantly more errors for non-collocates (e.g., *angry use*) than real collocations, demonstrating a tendency to falsely accept implausible adjective-noun pairs. They attributed this finding to high-type-frequency schematic frames (e.g., *strong + X*), which prompted participants to overgeneralize and accept even unattested combinations. Together, these studies underscore the influence of abstract schematic patterns in multiword processing. Extending this line of inquiry to L2 CMS processing, the present study examines how type frequency modulates such processing and its potential interactions with token frequency, L2 proficiency, and the phrasal status of CMSs.

Moreover, few studies to date have systematically compared the processing of phrasal versus non-phrasal CMSs (i.e., the constituency effect), and existing findings remain inconclusive. Within this limited body of research, Arnon and Cohen-Priva (2013) found a significant effect of multiword token frequency on duration for both phrases and non-phrases, with no evidence of a difference in the magnitude of the effect between the two types of sequences. This suggests that constituency—whether a sequence functions as a syntactic constituent—may be a less important feature of multiword sequence processing. A parallel finding was documented by Jolsvai et al. (2020) in their investigation involving native English speakers, further highlighting the limited role of constituency in sequence processing. However, other evidence suggests that non-phrases may not share the same representational status as phrases. For instance, Jolsvai et al. (2013) compared processing latencies for three types of multiword sequences in native speakers: idiomatic expressions (e.g., *in the wind*), compositional phrases (e.g., *had a dream*), and non-phrasal sequences (e.g., *know it gets*). Their results showed a consistent facilitatory effect of token frequency on processing efficiency across all three sequence types. However, while processing latencies for compositional phrases were comparable to those of frequency-matched idiomatic phrases, non-phrasal sequences exhibited significantly longer latencies than idiomatic phrases. This pattern suggests that phrasal sequences, despite their compositional structure, may be represented and processed similarly to idiomatic phrases, whereas non-phrasal sequences are not. These mixed findings highlight the need for further research to clarify the differences between phrase and non-phrase processing, and the potential impact of constituency on L2 learners at different proficiency levels.

Finally, L2 proficiency is also a key factor in multiword sequence processing. A growing body of research demonstrates that learners at different proficiency levels exhibit distinct processing patterns (e.g., Nekrasova, 2009; Shantz, 2017; Siyanova-Chanturia et al., 2011; Wolter & Yamashita, 2018). For instance, Siyanova-Chanturia et al.’s (2011) eye-tracking study revealed a significant interaction between phrase type (i.e., binomial versus reversed form) and L2 proficiency when comparing processing of high-token-frequency binomials (e.g., *bride and groom*) versus their low-token-frequency reversed forms (e.g., *groom and bride*). While more proficient L2 learners processed binomials significantly faster than reversed forms, less proficient L2 learners showed equivalent reading times for both forms. Shantz (2017) further demonstrated a proficiency-dependent token frequency effect using a self-paced reading task. Specifically, phrase token frequency modulated non-native grammaticality judgments, exhibiting a U-shaped relationship with L2 proficiency. This pattern suggests a developmental shift from reliance on phrase token frequency to abstraction of generalizable rules as linguistic experience accumulates. Likewise, Wolter and Yamashita (2018) found that while intermediate Japanese L2 learners relied more heavily on word frequency, advanced learners prioritized collocational frequency, demonstrating progression toward native-like processing strategies. Collectively, these findings underscore the necessity of accounting for L2 proficiency when examining how frequency (both token and type) and constituency effects operate in L2 CMS processing.

To address these research gaps, the present study investigates the effects of token frequency, type frequency, and constituency on L2 CMS processing among Chinese learners of English at two proficiency levels. Specifically, this study aims to answer the following research questions:

(1) To what extent does token frequency influence L2 phrasal and non-phrasal CMS processing, and how does this effect vary across L2 proficiency levels?

(2) To what extent does type frequency influence L2 phrasal and non-phrasal CMS processing, and how does this effect vary across L2 proficiency levels?

(3) To what extent does constituency (i.e., phrasal vs. non-phrasal) influence L2 CMS processing, and how does this effect vary across L2 proficiency levels?

Drawing on theoretical perspectives and empirical evidence, we formulated three key hypotheses. First, we predicted that both proficiency groups would process high-token-frequency phrasal and non-phrasal CMSs more efficiently (i.e., with faster reaction times and higher accuracy), with this advantage being potentially stronger in higher-proficiency learners due to their more entrenched CMS representations (e.g., Siyanova-Chanturia et al., 2011). Second, building on usage-based approaches (Bybee, 2008) and prior research (Matthews & Bannard, 2010), we hypothesized that high-type-frequency CMSs would be processed more efficiently than their low-type-frequency counterparts in both proficiency groups, and that the HP group would demonstrate more developed schematic knowledge than the LP group. Third, given that phrasal CMSs typically exhibit stronger internal associations and semantic relationships (Ellis et al., 2008; Nekrasova, 2009), we anticipated that they would be processed more efficiently than non-phrasal CMSs in both proficiency groups. However, we predicted this structural advantage (or constituency effect) might attenuate in higher-proficiency learners due to their advanced linguistic knowledge and greater flexibility in cognitive processing.

## 3. Method

To investigate the effects of token frequency, type frequency, and constituency on L2 CMS processing, as well as their potential variation across proficiency levels, an online phrasal decision task was employed (Arnon & Snider, 2010; Jolsvai et al., 2020; Yu et al., 2025).

### 3.1. Participants

Initially, 79 participants were recruited for the online phrasal decision task: 44 first-year and 35 third-year undergraduate English majors from a university in mainland China. All the participants were native Mandarin speakers with English as their L2. Before the experiment, they provided written informed consent (see Supplementary Table S1 for details), and completed the 60-point Quick Placement Test (QPT) (University of Cambridge Local Examinations Syndicate, 2002) for objective English proficiency assessment.

Following the completion of the phrasal decision task, participants with error rates (ERs) exceeding 25%, or exhibiting 15% extremely long or short response times (RTs) (≥5000 milliseconds (ms) or ≤400 ms, see Section 3.4) (N. Jiang & Nekrasova, 2007; Kosaka, 2024; Wolter & Gyllstad, 2013; Yu et al., 2025) were excluded from the analysis, as their data were considered inappropriate—they were either not engaging in the task or the task was too difficult for them. This resulted in the exclusion of 5 first-year and 3 third-year students, leaving a sample comprising 39 first-year and 32 third-year students. They were then divided into two groups based on their QPT scores. To ensure a clear distinction between proficiency groups and avoid score overlap, we excluded 11 participants with QPT scores in the 41–42 range^1^. The remaining 60 students were then divided into two groups: a higher proficiency (hereafter, HP) group, comprising 30 third-year students with the highest test scores (estimated to be at an upper-intermediate level based on QPT proficiency standards), and a lower proficiency (hereafter, LP) group, consisting of 30 first-year students with the lowest scores (estimated to be at a lower-intermediate level). An independent-samples *t*-test confirmed a statistically significant difference in English proficiency between the two groups (*t*(58) = 16.83, *p* < 0.001).

During the online phrasal decision experiment, participants completed a language background questionnaire to provide contextual information about their linguistic experiences. As summarized in Table 1, none of the participants reported prior experience of living or studying in an English-speaking country, thereby controlling for potential confounding variables related to immersion in an English-speaking environment. Additionally, all participants were right-handed with normal or corrected-to-normal vision to control for motor and visual performance variations.

### 3.2. Stimuli

Based on previous research (e.g., Biber, 2009), 120 tri-gram phrase frames with a single slot (e.g., from his *) were selected. This study computed several key metrics: type frequency and overall token frequency of each phrase frame, token frequency of each CMS, and slot entropy. Specifically, type frequency of a phrase frame refers to the total number of specific CMSs that exemplify the frame. Overall token frequency of a phrase frame represents the cumulative occurrences of all CMSs (e.g., *from his home*, *from his office*) that instantiate the phrase frame (e.g., from his *) in a corpus. Token frequency of a CMS is defined as the number of times a specific sequence (e.g., *from his home*) appears in a corpus. For example, if the phrase frame from his * accommodates 6148 specific CMSs, its type frequency is 6148. If these 6148 CMSs collectively occur 55,688 times in a corpus, the overall token frequency of the phrase frame is 55,688. Similarly, if the CMS *from his home* appears 1729 times, its token frequency is 1729. All frequency metrics and slot entropy calculations in this study were derived from the Corpus of Contemporary American English (COCA). During computation, sequences containing punctuation, or numerals were excluded, and only lexical items of the same part of speech were considered as valid slot fillers.

Although type frequency is directly correlated with the productivity of linguistic patterns, it has a notable limitation: it does not account for the frequency distribution of the words filling a given slot (Matthews & Bannard, 2010). For instance, consider two phrase frames, A and B, with identical type frequencies (e.g., 5) and overall token frequencies (e.g., 5000). However, the token frequencies of the five specific multiword sequences within A and B are 4996, 1, 1, 1, 1 and 1500, 1000, 1000, 1000, 500, respectively. In such cases, the productivity of phrase frame A may be compromised due to the high predictability of its most frequent slot filler (i.e., the word in the CMS with a token frequency of 4996). This skewed distribution likely results in the entrenchment of the high-token-frequency multiword sequence in the mental lexicon, overshadowing the schematic structure of its phrase frame and reducing the likelihood of the frame being recognized as a productive pattern in its own right. In contrast, the more balanced frequency distribution of slot fillers in phrase frame B facilitates its recognition as a productive schematic pattern, enhancing its entrenchment and generalizability. To better capture the frequency distribution of slot fillers and the productivity of phrase frames, this study employed Shannan and Weaver’s (1949) formula to calculate slot entropy (Gries & Ellis, 2015; Matthews & Bannard, 2010).

(1) $$H(X)=\sum_{x\in X}p(x)\log_{2}p(x)$$

In this formula, *X* denotes a slot within a phrase frame, *x* represents a specific lexical item occupying that slot, and *p*(*x*) signifies the probability of observing each *x* in the slot. Applying this formula, the slot entropies for the two illustrative phrase frames A and B were calculated as 0.01 and 2.25, respectively. A higher slot entropy value reflects greater uncertainty regarding potential lexical fillers for a given slot, thereby indicating a higher likelihood of the phrase frame being perceived as a productive schematic pattern (Matthews & Bannard, 2010). The present study used the Perl programming language to calculate slot entropy values for phrase frames. This metric provided a refined measure of type frequency, ensuring that high-type-frequency frames were more productive, while low-type-frequency frames were less productive.

This study operationalized phrase frame productivity using two established metrics from the literature (Gries & Ellis, 2015; Matthews & Bannard, 2010): type frequency and slot entropy. High productive frames were defined as those exhibiting both higher type frequency and greater slot entropy, while low productive frames showed lower values on both metrics. As no established thresholds exist for classifying frame productivity, we empirically determined cut-off values by analyzing the distribution of type frequencies and entropies across all frames in our dataset. The resulting classification cutoffs were 4000 for type frequency and 7 for entropy. Frames meeting both thresholds (type frequency ≥ 4000 and entropy ≥ 7) were classified as high productive; those below both thresholds (type frequency < 4000 and entropy < 7) as low productive. Based on these criteria, four groups of frames were selected: Group A comprised 11 high-type-frequency phrasal frames (e.g., from his *), Group B comprised 11 high-type-frequency non-phrasal frames (e.g., with * of), Group C included 11 low-type-frequency phrasal frames (e.g., at any *), and Group D included 11 low-type-frequency non-phrasal frames (e.g., at * of). The slot fillers of these frames were content words constrained by a uniform part of speech but generally unrestricted by slot semantics. Table 2 displays the descriptive statistics of productivity metrics across phrase frame groups. The type frequency values were log10-transformed.

One-way ANOVAs revealed significant differences among the four groups of phrase frames in both log-transformed type frequency (*F*(3, 40) = 24.64, *p* < 0.001) and slot entropy (*F*(3, 40) = 30.60, *p* < 0.001). Post hoc analyses indicated that, for both measures, there were significant differences between any group from the high productive sets (i.e., Groups A and B) and any group from the low productive sets (i.e., Groups C and D) (all *p* ≤ 0.001). However, no significant differences were found within each set of groups (i.e., between Groups A and B or between Groups C and D). For the full list of the four groups of phrase frames used in the experiment, please refer to Supplementary Table S2.

Then, for the 22 high-type-frequency phrase frames (i.e., Groups A and B), 44 CMSs were selected based on their token frequency and divided into four groups: 11 phrasal CMSs (e.g., *from his home*) with high type frequency and high token frequency (Group 1), 11 non-phrasal CMSs (e.g., *with members of*) with high type frequency and high token frequency (Group 2), 11 phrasal CMSs (e.g., *from his bed*) with high type frequency and low token frequency (Group 3), and 11 non-phrasal CMSs (e.g., *with evidence of*) with high type frequency and low token frequency (Group 4). Similarly, for the 22 low-type-frequency phrase frames (i.e., Groups C and D), 44 CMSs were selected and divided into four groups: 11 phrasal CMSs (e.g., *at any moment*) with low type frequency and high token frequency (Group 5), 11 non-phrasal CMSs (e.g., *at risk of*) with low type frequency and high token frequency (Group 6), 11 phrasal CMSs (e.g., *at any minute*) with low type frequency and low token frequency (Group 7), and 11 non-phrasal CMSs (e.g., *at times of*) with low type frequency and low token frequency (Group 8).

The eight groups of CMSs were carefully matched for comparability. Consistent with methodological approaches in prior studies (e.g., N. Jiang & Nekrasova, 2007; Siyanova-Chanturia et al., 2011; Wolter & Yamashita, 2018), we set high token frequency as more than or equal to 1000 occurrences and low token frequency as fewer than or equal to 310 occurrences in COCA, thereby maintaining the significant frequency differential necessary for experimental contrast. Table 3 details the statistical characteristics for all eight CMS groups. All frequency measures were log10 transformed. One-way ANOVAs revealed no significant differences among the eight groups in mean word length (*F*(7, 256) = 0.28, *p* > 0.05), mean log-transformed word token frequency (*F*(7, 256) = 0.61, *p* > 0.05), or mean log-transformed bi-gram token frequency (*F*(7, 168) = 0.38, *p* > 0.05). However, significant differences were observed in log-transformed whole-string token frequency (*F*(7, 80) = 65.47, *p* < 0.001). Post hoc analyses indicated significant differences between any group from the high-token-frequency sets (i.e., Groups 1, 2, 5, 6) and any group from the low-token-frequency sets (i.e., Groups 3, 4, 7, 8) (all *p* < 0.001), but no significant differences within each set of groups. For the complete list of the eight groups of CMSs used in the experiment, please refer to Supplementary Table S3.

To elicit negative responses, 88 grammatically erroneous word strings (e.g., *view own my*, *in have common*) were used as interference items. The experimental materials thus comprised 88 experimental items and 88 interference items. In the formal experiment, 176 items (88 experimental items and 88 interference items) were divided into two blocks: Block A (including CMSs in Groups 1, 4, 6, 7) and Block B (including CMSs in Groups 2, 3, 5, 8). Following Supasiraprapa (2019), this experiment employed a within-subject counterbalanced design. All participants completed both experimental blocks. Specifically, half of each proficiency group were randomly assigned to complete Block A first and then Block B, while the other half completed Block B first and then Block A. This counterbalancing procedure controlled for the potential block order effect on the experimental results. Between the two blocks, there was a break during which participants completed a language background questionnaire to mitigate repetition effects before proceeding to the second block.

### 3.3. Procedures

A phrasal decision task was administered. In this task, participants were instructed to judge, as quickly and accurately as possible, whether the presented item could form part of an English sentence, by pressing “J” for “Yes” or “F” for “No”. The experiment began with an instruction (written in Chinese), followed by a practice session designed to familiarize participants with the experimental procedure, and then two test sessions separated by an interim break.

All items were presented to the participants in a personalized randomized order using E-Prime 2.0. Each trial ran in the following procedure: a red fixation point “+” appeared on the center of the screen for 1000 milliseconds to remind participants that a trial began; then, a test item showed up on the screen; participants made a judgment and pressed the corresponding key (“J” or “F”) as quickly and accurately as possible. Then the next trail started. Participants’ RTs and ERs were recorded. The experiment took approximately 12–15 min to complete.

### 3.4. Data Analysis

For the RT data analysis, all incorrect responses were excluded. As in Kosaka (2024), RTs that were three standard deviations from each participant’s mean were removed as outliers. In addition, while following established cut-off criteria from CMS processing studies with highly proficient L2 learners (e.g., 400–4000 ms in N. Jiang & Nekrasova, 2007), we adjusted our thresholds to account for two critical factors: the inclusion of low-frequency (in terms of both token and type frequency) and non-phrasal items in our stimulus set, and the comparatively lower proficiency levels of our participants. We decided to set the low cut-off at 400 ms and high cut-off 5000 ms. Therefore, RTs shorter than 400 ms or longer than 5000 ms were excluded. This procedure resulted in a 4.39% data loss for the HP group and a 9.47% data loss for the LP group. Table 4 presents the means and standard deviations of log10-transformed RTs (LogRTs) and ERs (in percent) across the eight sequence conditions.

In line with previous studies (e.g., Chen et al., 2023; N. Jiang & Nekrasova, 2007), we conducted separate 2 × 2 × 2 repeated-measures ANOVAs (by participants)^2^ on mean log-transformed RTs and ERs for each proficiency group, using the General Linear Model-Repeated Measures procedure in SPSS (version 26.0). These analyses examined overall processing patterns with token frequency (high, low), type frequency (high, low), and constituency (phrase, non-phrase) as within-subjects factors.

To directly test specific hypotheses, we conducted planned paired-samples *t*-tests (Bonferroni-corrected) rather than post hoc analyses. These planned comparisons could isolate each target factor by controlling for other variables (e.g., token frequency effects tested only within identical type frequency and structure conditions), and maintain direct alignment with prior literature, ensuring that our analysis targets the same effects documented in previous studies. Specifically, to investigate token frequency effects, we compared high- versus low-token-frequency conditions while holding constant the other two factors (i.e., type frequency and constituency). Therefore, we compared the following groups: Group 1 (high type, high token, phrase) versus Group 3 (high type, low token, phrase), Group 2 (high type, high token, non-phrase) versus Group 4 (high type, low token, non-phrase), Group 5 (low type, high token, phrase) versus Group 7 (low type, low token, phrase), and Group 6 (low type, high token, non-phrase) versus Group 8 (low type, low token, non-phrase). For type frequency effects, we contrasted high- versus low-type-frequency groups with matched token frequency and constituency. The comparison groups were: Group 1 versus Group 5, Group 2 versus Group 6, Group 3 versus Group 7, and Group 4 versus Group 8. Constituency effects were assessed by comparing phrasal versus non-phrasal sequences within identical frequency conditions. Therefore, we compared Group 1 and Group 2, Group 3 and Group 4, Group 5 and Group 6, and Group 7 and Group 8. The planned comparisons employed paired-samples *t*-tests with Bonferroni correction (adjusted *α* = 0.0042 based on 12 comparisons).

## 4. Results

### 4.1. General Processing Patterns

For the HP group, the repeated measures ANOVA on log-transformed RTs showed significant main effects of token frequency (*F*(1, 29) = 42.21, *p* < 0.001, *ƞ*^2^ = 0.59), type frequency (*F*(1, 29) = 44.29, *p* < 0.001, *ƞ*^2^ = 0.60), and constituency (*F*(1, 29) = 46.51, *p* < 0.001, *ƞ*^2^ = 0.62). The repeated measures ANOVA on ERs also revealed significant main effects of token frequency (*F*(1, 29) = 28.52, *p* < 0.001, *ƞ*^2^ = 0.50), type frequency (*F*(1, 29) = 13.83, *p* = 0.001, *ƞ*^2^ = 0.32), and constituency (*F*(1, 29) = 17.31, *p* < 0.001, *ƞ*^2^ = 0.37).

For the LP group, the repeated measures ANOVA on log-transformed RTs demonstrated significant main effects of token frequency (*F*(1, 29) = 40.38, *p* < 0.001, *ƞ*^2^ = 0.58), type frequency (*F*(1, 29) = 24.26, *p* < 0.001, *ƞ*^2^ = 0.46), and constituency (*F*(1, 29) = 76.53, *p* < 0.001, *ƞ*^2^ = 0.73). Moreover, a marginally significant three-way interaction was found between type frequency, token frequency, and constituency (*F*(1, 29) = 3.05, *p* = 0.09, *ƞ*^2^ = 0.10), indicating that constituency status (i.e., phrasal vs. non-phrasal) might modulate frequency effects. The repeated measures ANOVA on ERs revealed significant main effects of token frequency (*F*(1, 29) = 30.97, *p* < 0.001, *ƞ*^2^ = 0.52), type frequency (*F*(1, 29) = 19.49, *p* < 0.001, *ƞ*^2^ = 0.40), and constituency (*F*(1, 29) = 15.96, *p* < 0.001, *ƞ*^2^ = 0.36). In addition, a significant two-way interaction between token frequency and constituency (*F*(1, 29)= 24.64, *p* < 0.001, *ƞ*^2^ = 0.46) was found, indicating that the effect of token frequency on CMS processing accuracy was contingent upon phrasal status.

These results indicate that the two proficiency groups processes CMSs more efficiently when those sequences occur more frequently (i.e., token frequency effect), belong to more productive schematic patterns (i.e., type frequency effect), and constitute complete phrasal units (i.e., constituency effect). However, the magnitude and interaction patterns of these effects differed substantially between groups. Notably, the LP group exhibited particularly strong effects of constituency (*ƞ*^2^ = 0.73 for log-transformed RTs) and more complex interaction patterns in their data. To precisely characterize these effects, we conducted planned paired-samples *t*-tests with Bonferroni correction (adjusted *α* = 0.0042). Results are reported in the following three subsections, with all analyses reporting *t*-values, *p*-values, and Cohen’s *d* effect sizes.

### 4.2. Token Frequency Effects

For the HP group, the paired-samples *t*-tests showed that, under the condition of high type frequency, this group only processed non-phrasal CMSs with high token frequencies (Group 2) significantly faster than non-phrasal CMSs with low token frequencies (Group 4) (*t* = −3.55, *p* = 0.001, *d* = −0.65). Under the condition of low type frequency, this group processed phrasal CMSs with high token frequencies (Group 5) significantly faster than phrasal CMSs with low token frequencies (Group 7) (*t* = −5.26, *p* < 0.001, *d* = −0.96), and processed non-phrasal CMSs with high token frequencies (Group 6) significantly faster than non-phrasal CMSs with low token frequencies (Group 8) (*t* = −5.42, *p* < 0.001, *d* = −0.99). In terms of ERs, the results showed that, only under the condition of low type frequency, this group made significantly fewer errors on phrasal CMSs in Group 5 than on phrasal CMSs in Group 7 (*t* = −3.50, *p* = 0.002, *d* = −0.64).

For the LP group, the results indicated that, only under the condition of high type frequency, this group processed phrasal CMSs with high token frequencies (Group 1) significantly faster than phrasal CMSs with low token frequencies (Group 3) (*t* = −3.93, *p* < 0.001, *d* = −0.72), and processed non-phrasal CMSs with high token frequencies (Group 2) significantly faster than non-phrasal CMSs with low token frequencies (Group 4) (*t* = −3.76, *p* = 0.001, *d* = −0.69). The ER results showed that the LP group committed significantly fewer errors on Group 2 non-phrasal CMSs than Group 4 (*t* = −3.75, *p* = 0.001, *d* = −0.69) and on Group 6 non-phrasal CMSs than Group 8 (*t* = −4.42, *p* < 0.001, *d* = −0.81).

The results above demonstrate that token frequency significantly influenced the two proficiency groups’ phrasal and non-phrasal CMS processing. However, this effect appeared to differ between the two proficiency groups: the influence of token frequency was more pronounced in the HP group’s processing of both phrasal and non-phrasal CMSs with low type frequencies, whereas it was identified in the LP group’s processing of phrasal and non-phrasal CMSs with high type frequencies.

### 4.3. Type Frequency Effects

Under the condition of low token frequency, the HP group exhibited a significant processing advantage for phrasal CMSs with high type frequencies (Group 3) over phrasal CMSs with low type frequencies (Group 7) (*t* = −3.16, *p* = 0.004, *d* = −0.58), and for non-phrasal CMSs with high type frequencies (Group 4) over non-phrasal CMSs with low type frequencies (Group 8) (*t* = −4.95, *p* < 0.001, *d* = −0.90). In terms of ERs, results showed that the HP group made fewer errors on CMSs in Group 3 than CMSs in Group 7 (*t* = −3.40, *p* = 0.002, *d* = −0.62).

For the LP group, the results showed that under the condition of high token frequency, this group processed phrasal CMSs with high type frequencies (Group 1) significantly faster than phrasal CMSs with low type frequencies (Group 5) (*t* = −3.30, *p* = 0.003, *d* = −0.60); and they also processed non-phrasal CMSs with high type frequencies (Group 2) significantly faster than non-phrasal CMSs with low type frequencies (Group 6) (*t* = −3.75, *p* = 0.001, *d* = −0.68). In terms of ERs, the LP group made significantly fewer errors on phrasal CMSs in Group 1 over phrasal CMSs in Group 5 (*t* = −4.01, *p* < 0.001, *d* = −0.73) and on phrasal CMSs in Group 3 over phrasal CMSs in Group 7 (*t* = −3.46, *p* = 0.002, *d* = −0.63).

These results indicated that type frequency also had an impact on the two proficiency groups’ CMS processing. However, notable differences emerged in type frequency effects across proficiency levels: for the HP group, the type frequency advantage was observed specifically in the processing of low-token-frequency CMSs (both phrasal and non-phrasal), while for the LP group, this effect was more evident in the processing of high-token-frequency CMSs (both phrasal and non-phrasal).

### 4.4. Constituency Effects

The HP group exhibited differential constituency effects based on type frequency conditions. Under the condition of high type frequency, the HP group only processed low-token-frequency phrasal CMSs (Group 3) significantly faster than low-token-frequency non-phrasal CMSs (Group 4) (*t* = −4.39, *p* < 0.001, *d* = −0.80). Under the condition of low type frequency, processing advantages emerged for both high-token-frequency phrasal (Group 5 versus Group 6: *t* = −5.35, *p* < 0.001, *d* = −0.98) and low-token-frequency phrasal CMSs (Group 7 versus Group 8: *t* = −6.08, *p* < 0.001, *d* = −1.11). For ERs, a significant phrasal advantage only appeared in the high-type-frequency condition (Group 3 versus Group 4: *t* = −4.27, *p* < 0.001, *d* = −0.78).

The LP group demonstrated significant constituency effects in processing speed across all comparison groups: Group 1 versus Group 2 (*t* = −5.46, *p* < 0.001, *d* = −1.00), Group 3 versus Group 4 (*t* = −5.14, *p* < 0.001, *d* = −0.94), Group 5 versus Group 6 (*t* = −5.81, *p* < 0.001, *d* = −1.06), and Group 7 versus Group 8 (*t* = −4.01, *p* < 0.001, *d* = −0.73). For ERs, significant constituency effects emerged under the low-token-frequency condition, with fewer errors on phrasal CMSs in Group 3 than on non-phrasal CMSs in Group 4 (*t* = −4.60, *p* < 0.001, *d* = −0.78), and fewer errors on phrasal CMSs in Group 7 than on non-phrasal CMSs in Group 8 (*t* = −3.13, *p* = 0.004, *d* = −0.57).

The results above demonstrate a robust constituency effect on CMS processing across both proficiency groups. Notably, the data suggested that this effect was more pronounced in the LP group, highlighting the differential impact of constituency on L2 CMS processing efficiency.

## 5. Discussion

### 5.1. The Effect of Token Frequency in L2 CMS Processing

As expected, both proficiency groups exhibited token frequency effects in their CMS processing, with high-token-frequency sequences being processed significantly faster and with greater accuracy than their low-token-frequency counterparts. To enable direct comparison with prior findings, we analyze these effects through separate examinations of phrasal and non-phrasal CMSs.

For phrasal CMS processing, distinct patterns in token frequency effects were observed between the two proficiency groups. These findings align with a growing body of research that highlights the differential processing patterns exhibited by L2 learners across distinct proficiency levels (e.g., Siyanova-Chanturia et al., 2011; Wolter & Yamashita, 2018). Specifically, while less proficient learners showed a significant token frequency effect only for high-type-frequency phrases, more proficient learners demonstrated this effect specifically for low-type-frequency phrases. This double dissociation suggests that the influence of token frequency is dynamically modulated by the interaction between L2 proficiency and type frequency. These findings may help explain inconsistencies in prior phrase processing studies (e.g., Arnon & Cohen-Priva, 2013; Chen et al., 2023; Jeong & Jiang, 2019; Jolsvai et al., 2020; Supasiraprapa, 2019; Valsecchi et al., 2013) by demonstrating how L2 proficiency levels and stimulus type frequency profiles modulate token frequency effects.

In addition, the present study identifies significant token frequency effects in the processing of non-phrasal sequences across both proficiency groups. This finding is consistent with prior research that documented processing advantages for non-phrasal CMSs (e.g., Arnon & Cohen-Priva, 2013; Tremblay & Baayen, 2010; Tremblay et al., 2011). However, it diverges from the findings of Jeong and Jiang (2019), who observed token frequency effects exclusively in phrasal sequence processing for both L1 and L2 speakers, with no comparable effects for non-phrasal sequences. Based on their results, Jeong and Jiang (2019) proposed that structurally incomplete sequences may lack coherent semantic integrity, which could hinder the establishment of form-meaning associations and their storage in the mental lexicon. The discrepancy between their findings and those of the present study may be attributed to differences in stimulus characteristics. In Jeong and Jiang’s study, low-token-frequency non-phrases (e.g., *did not appear*, *be more likely*, *it would be noted*) appeared more acceptable and realistic than low-token-frequency phrases (e.g., *like the whole*, *to other words*, *both the way*). This may have facilitated faster processing of low-token-frequency non-phrases, thereby reducing the observed processing differences between high- and low-token-frequency non-phrases. Building on Jeong and Jiang’s (2019) work, the present study suggests that the meanings of multiword sequences may exist along a continuous spectrum rather than as a strictly dichotomous construct, regardless of their structural completeness. This perspective allows for the possibility that CMSs with even partial semantic coherence can exhibit psychological reality, even if they are structurally incomplete.

The observed token frequency effects in both phrasal and non-phrasal CMS processing imply that structural completeness may not necessarily serve as a prerequisite for mental representation. Rather, it appears that L2 learners establish form-meaning mappings for both high-token-frequency phrasal and non-phrasal sequences along a continuum characterized by varying degrees of semantic coherence. This perspective is consistent with usage-based approaches (e.g., Diessel, 2015; Ellis, 2002), which highlights the gradational and experience-driven nature of language acquisition and representation.

### 5.2. The Effect of Type Frequency in L2 CMS Processing

Consistent with our hypotheses, both proficiency groups demonstrated significant type frequency effects during CMS processing, with high-type-frequency CMSs being processed faster and more accurately than their low-type-frequency ones under matched conditions. In this section, type frequency effects are examined through separate analyses of low- and high-token-frequency CMSs.

The present study operationalized low-token-frequency CMSs as sequences occurring fewer than 310 times in COCA, a frequency threshold indicative of limited exposure opportunities for L2 learners. Such infrequent occurrence suggests that learners may be less likely to have established direct form-meaning mappings for these sequences, potentially leading to a greater reliance on schematic knowledge during online processing. Nonetheless, under the condition of low token frequency, this study revealed significant type frequency effects in the HP group’s processing speed and accuracy of both phrasal and non-phrasal CMSs, and a significant type frequency effect in the LP groups’ processing accuracy of phrasal CMSs. These findings corroborate and extend previous research. Specifically, Matthews and Bannard’s (2010) study revealed that L1 children demonstrated greater efficiency in producing unfamiliar CMSs when these sequences were embedded in highly productive schemas. Similarly, Wolter and Gyllstad (2013) found that schematic frames with high type frequency facilitated the interpretation of novel two-word combinations. The observed parallel between L1 and L2 processing patterns suggests that the cognitive mechanisms underlying schema (i.e., phrase frame) abstraction and application may be fundamentally similar across different language learning contexts, though potentially modulated by proficiency-related factors in L2 acquisition.

The current findings thus provide empirical support for the central tenet of usage-based theory (Bybee, 2008; Wolter & Gyllstad, 2013), which posits that higher type frequency leads to the establishment of stronger mental representations of productive schemas, thereby facilitating the processing of corresponding low-token-frequency linguistic items. In the context of the present study, it seems possible that phrase frames with higher type frequencies may develop stronger psychological representations through cumulative exposure to diverse exemplars, making them more readily available for the analysis and interpretation of novel or low-token-frequency CMSs. In contrast, phrase frames with lower type frequencies, instantiated through fewer and less varied exemplars, may develop weaker mental representations that are less accessible for application to infrequent CMSs.

Furthermore, the present study revealed a noteworthy finding: even under the condition of high token frequency, type frequency still significantly influenced the processing of phrasal and non-phrasal CMSs in the LP group. This finding suggests that the influence of type frequency is not limited to low-token-frequency CMSs but operates in a gradient manner across different token frequency ranges. However, no significant type frequency effects were observed in the HP group’s processing of high-token-frequency CMSs. This divergence suggests that as proficiency increases, learners tend to rely more on specific linguistic items and less on abstract linguistic patterns, reflecting a shift from schema-based to item-based processing.

This discovery extends existing theoretical understanding of the relationship between type frequency and token frequency. While existing theories emphasize the distinct roles of token frequency (i.e., reinforcing specific linguistic items, such as a CMS) and type frequency (i.e., consolidating abstract linguistic patterns, such as a phrase frame) in the representation of linguistic units within the mental lexicon (Bybee, 2008), the current study suggests that the relationship between the two is dynamic and interdependent: type frequency remains influential even for high-token-frequency sequences among less proficient learners, while more advanced learners show greater reliance on token frequency. This suggests a developmental progression in which the relative weighting of these frequency effects shifts with increasing proficiency.

### 5.3. The Effect of Constituency in L2 CMS Processing

The findings of this study provide robust evidence for the significant role of constituency in L2 CMS processing across both proficiency groups, with phrasal CMSs showing faster and more accurate processing than non-phrasal ones.

For the LP group, phrasal CMSs were processed significantly more efficiently than non-phrasal CMSs across all type and token frequency conditions. This consistent advantage demonstrates that structural integrity plays a critical role in L2 CMS processing for lower proficiency learners. The HP group exhibited a more nuanced pattern of constituency effects. While generally sensitive to phrasal structure, they processed high-frequency (both token and type) phrasal and non-phrasal CMSs equally efficiently. This indicates that, as learners attain higher proficiency, non-phrasal CMSs may achieve a representational and processing status comparable to their phrasal counterparts, provided that both token frequency and type frequency are sufficiently high.

One plausible explanation for the differential constituency effects observed in the two proficiency groups’ processing of high-token-frequency CMSs lies in the inherent salience of phrasal sequences. As noted by Arnon and Cohen-Priva (2013, p. 351), a phrasal CMS (e.g., *at any moment*) evokes a complete semantic event, forms a syntactic constituent, and is consistently produced as a single intonational unit, while a non-phrasal CMS (e.g., *an answer to*), despite having comparable sequence token frequency, is less semantically complete, crosses syntactic boundaries, and does not form a coherent intonational unit. This distinction indicates that phrasal sequences possess greater psychological salience than non-phrasal sequences (e.g., Ellis & Simpson-Vlach, 2009; Nesi & Basturkmen, 2006). As a result, structurally incomplete CMSs often fail to capture the attention of L2 learners with limited linguistic experience, which may explain why lower-proficiency learners encounter difficulties in processing non-phrasal sequences efficiently. However, as learners’ proficiency increases, they begin to recognize the recurrent nature of non-phrasal sequences and subsequently consolidate and strengthen their form-meaning associations in memory. Over time, these non-phrasal sequences may become more entrenched in learners’ mental representations, potentially reaching a level of processing efficiency comparable to that of phrasal items.

The present findings contribute to a growing yet limited body of research that has produced mixed results on the role of constituency in CMS processing. For example, Arnon and Cohen-Priva’s (2013) production study with native speakers found no significant constituency effect in the phonetic duration of phrases compared to non-phrases. In contrast, Jolsvai et al. (2013) revealed a fundamental distinction in the mental representation of phrasal and non-phrasal CMSs: phrasal CMSs showed evidence of holistic storage, whereas non-phrasal CMSs appeared to lack such integrated representation. The discrepancy between these studies and the present findings may stem from two key factors: differences in participant proficiency levels and variations in the type frequency of the research materials. Specifically, the current study highlights the developmental trajectory of L2 CMS processing, wherein the influence of constituency diminishes as learners gain proficiency, particularly when non-phrasal sequences exhibit high type frequencies. This suggests that type frequency acts as a moderating factor, enabling learners to overcome the processing challenges typically associated with non-phrasal structures.

## 6. Conclusions

This study employed an online phrasal decision task to examine the effects of token frequency, type frequency, and constituency on the processing of L2 CMSs by Chinese learners of English at two proficiency levels. Results revealed significant effects of these factors and their complex interactions. Both proficiency groups exhibited a token frequency effect in their L2 phrasal and non-phrasal CMS processing, indicating that both sequence types hold psychological reality in L2 learners’ mental representations. However, this effect was moderated by an interaction between type frequency and L2 proficiency, highlighting the dynamic nature of token frequency in L2 CMS processing. In addition, the higher-proficiency group exhibited type frequency effects only for low-token-frequency CMSs, whereas the lower-proficiency group mainly showed such effects for high-token-frequency CMSs. These findings suggest that the influence of type frequency operates on a continuum, with its role modulated by learners’ proficiency levels. Furthermore, both groups processed phrasal CMSs more efficiently than non-phrasal CMSs across nearly all frequency conditions, indicating a robust constituency effect. These findings collectively highlight the nuanced interplay between frequency, proficiency, and structural properties in L2 CMS processing.

The findings on L2 CMS processing have pedagogical implications. This study demonstrates token frequency effects in both phrasal and non-phrasal CMS processing, indicating that L2 instruction should prioritize not only high-token-frequency phrases but also non-phrasal sequences. To this end, L2 educators should emphasize the importance of compositional expressions in language teaching by raising learners’ awareness of the phraseological nature of these sequences and their critical role in achieving native-like fluency and idiomaticity. This study also underscores the crucial role of type frequency in L2 CMS processing. Considering that learners develop relatively weak mental representations of low-type-frequency phrase frames, targeted explicit instruction should be implemented to increase their perceptual salience, thereby facilitating the simultaneous internalization and automatization of both CMS and schema knowledge. Furthermore, this study highlights the importance of constituency in the processing and representation of L2 CMSs; however, it also reveals that non-phrasal CMSs can achieve similar representational status to phrasal ones when L2 proficiency is sufficiently high. Therefore, it is crucial for L2 educators to adopt a proficiency-sensitive approach to teaching CMSs. For lower-proficiency learners, instruction should focus on high-token-frequency and high-type-frequency phrasal CMSs, as these sequences align more closely with the hierarchical structures that facilitate cognitive processing, making them easier for learners to internalize. As learners progress to higher proficiency levels, educators can gradually introduce non-phrasal CMSs and provide targeted practice to help learners internalize their structures and meanings.

While offering novel insights into L2 CMS processing, this study has several limitations. The small sample size of 11 stimuli per sequence condition, incomplete matching of slot positions and syntactic categories across the four phrase frame groups, and exclusive focus on the online comprehension of L2 CMSs may limit the generalizability of the findings. Future research could address these limitations by employing larger and more balanced stimulus sets, ensuring rigorous matching of slot positions and syntactic categories, and incorporating multi-method approaches (e.g., combining real-time processing measures with off-line production tasks) to enable methodological triangulation of different aspects of CMS representation and processing. Such efforts would provide a more comprehensive understanding of how L2 CMSs are processed, represented, and acquired.

## Figures and Tables

**Table 1:** Biographical data for participants (standard deviation in parentheses).

Proficiency	N	Age	Gender (M/F)	YFEE	YREC	QPT Score
HP	30	20.67 (0.55)	5/25	12.73 (0.52)	0	45.07 (1.89)
LP	30	18.43 (0.50)	7/23	10.47 (0.51)	0	36.20 (2.31)

Note: N = number of participants; YFEE = years of formal English education; YREC = years of residence in an English-speaking country.

**Table 2:** Mean log-transformed type frequency and entropy across phrase frame groups.

Group	Log-Transformed Type Frequency		Entropy	
	Mean	SD	Mean	SD
Group A	3.72	0.10	8.23	0.68
Group B	3.84	0.21	8.41	0.92
Group C	3.25	0.29	5.62	1.14
Group D	3.20	0.22	5.89	0.75

**Table 3:** Mean length and log-transformed token frequency for the eight sequence conditions.

Group	Word Length		Log-Transformed Word Token Frequency		Log-Transformed Bi-Gram Token Frequency		Log-Transformed Whole-String Token Frequency	
	Mean	SD	Mean	SD	Mean	SD	Mean	SD
Group 1	4.18	2.13	6.13	0.99	4.41	0.59	3.35	0.28
Group 2	4.09	2.39	6.41	1.00	4.32	0.71	3.39	0.28
Group 3	4.03	2.08	6.13	1.00	4.24	0.78	2.31	0.10
Group 4	4.03	2.38	6.42	1.01	4.17	0.84	2.31	0.10
Group 5	3.76	1.44	6.17	0.92	4.34	0.60	3.36	0.29
Group 6	3.79	1.85	6.24	0.89	4.19	0.58	3.39	0.25
Group 7	3.88	1.52	6.14	0.96	4.26	0.71	2.24	0.23
Group 8	3.67	1.78	6.36	0.74	4.17	0.48	2.24	0.30

**Table 4:** Means of LogRTs and ERs with their standardized deviations in parentheses.

LogRTs/ERs	Proficiency	Group 1	Group 2	Group 3	Group 4	Group 5	Group 6	Group 7	Group 8
LogRTs	HP	3.11 (0.09)	3.14 (0.08)	3.13 (0.11)	3.19 (0.09)	3.12 (0.08)	3.18 (0.09)	3.17 (0.09)	3.24 (0.08)
	LP	3.15 (0.09)	3.22 (0.07)	3.20 (0.09)	3.27 (0.09)	3.20 (0.07)	3.27 (0.08)	3.24 (0.08)	3.28 (0.09)
ERs	HP	1.52 (3.45)	3.64 (4.53)	2.12 (3.91)	7.58 (5.89)	3.03 (4.36)	6.36 (6.38)	6.97 (5.69)	10.30 (9.47)
	LP	2.73 (4.24)	5.15 (4.58)	3.03 (4.36)	11.51 (9.53)	7.88 (7.05)	7.58 (7.95)	8.18 (7.30)	15.46 (10.44)

## Data Availability

The datasets generated during and/or analyzed during the current study are available from the corresponding author upon reasonable request.

## Notes

1 We conducted supplementary analyses to assess the robustness of our findings after including participants initially excluded based on QPT scores. The results remained largely consistent with the original analyses, with all key effects retaining significance and demonstrating comparable effect sizes.

2 Repeated-measures ANOVAs conducted by items on log-transformed RTs and ERs produced results largely consistent with the analyses conducted by participants.

## Supplementary Materials

The following supporting information can be downloaded at: https://www.mdpi.com/article/10.3390/bs15060734/s1, Table S1: Informed Consent Form; Table S2: List of the Four Groups of Phrase Frames Used in Phrasal Decision Task; Table S3: List of the Eight Groups of CMSs Used in Phrasal Decision Task.
