# Supplementary material for: Representation and Processing of L2 Compositional Multiword Sequences: Effects of Token Frequency, Type Frequency, and Constituency
Source: Behav Sci (Basel). 2025 May 26;15(6):734. doi: 10.3390/bs15060734 (PMC12189086; doi:10.3390/bs15060734)
Supplement: Supplementary file 1 [file behavsci-15-00734-s001.zip › behavsci-3550591-supplementary.pdf]

Table S1: Informed Consent Form

本人自愿参与本实验。我理解并同意以下事项：

1. 我的个人信息将被严格保密，有关我的实验数据仅研究者可见。
2. 我的实验数据将以匿名方式处理，使用编号标识，不会在任何公开或内部文件中透露我的真实姓名或其他可识别信息。
3. 我有权在实验的任何阶段无条件退出，且不会因此受到任何不利影响。

签名：_________

日期：_________

[I voluntarily agree to participate in this experiment. I understand and agree to the following:

1. My personal information will be kept strictly confidential and will only be accessible to the researchers.
2. My data will be anonymized and identified by a unique code, ensuring that my name or any other identifiable information will not be disclosed in any public or internal documents.
3. I retain the right to withdraw from the experiment at any stage without any adverse consequences.

Signature: _________

Date: _________]

Table S2: List of the Four Groups of Phrase Frames Used in Phrasal Decision Task

Group A	Group B	Group C	Group D
after a *	for * of	in other *	in * with
from his *	with * of	along the *	at * with
within the *	in * for	on one *	to what *
with their *	a * of	at any *	at * of
on their *	the * from	by all *	high * of
as an *	the * between	in any *	an * with
a large *	for * in	the early *	an * to
the main *	* in which	the past *	* to which
to * them	* for the	take a NOUN	* away from
make a *	be * in	tell the *	would * to
to * it	if you *	* to life	they * what

Table S3: List of the Eight Groups of CMSs Used in Phrasal Decision Task

**Group 1**	**Group 2**	**Group 3**	**Group 4**
after a while	for thousands of	after a fight	for dozens of
from his home	with members of	from his bed	with evidence of
within the context	in prison for	within the park	in love for
with their families	a period of	with their problems	a role of
on their feet	the data from	on their arms	the scene from
as an excuse	the connection between	as an invitation	the conversation between
a large amount	for women in	a large screen	for months in
the main reason	situation in which	the main concern	places in which
to protect them	wait for the	to believe them	arrested for the
make a difference	be included in	make a video	be said in
to break it	if you live	to want it	if you realize
**Group 5**	**Group 6**	**Group 7**	**Group 8**
in other states	in agreement with	in other sports	in business with
along the river	at war with	along the ground	at work with
on one side	to what extent	on one person	to what kind
at any moment	at risk of	at any minute	at times of
by all means	high cost of	by all sorts	high quality of
in any case	an interview with	in any part	an end with
the early days	an answer to	the early summer	an act to
the past century	degree to which	the past years	attention to which
take a shower	run away from	take a rest	go away from
tell the story	would try to	tell the public	would learn to
come to life	they know what	return to life	they see what
